# Supplementary material for: Genome of the estuarine oyster provides insights into climate impact and adaptive plasticity
Source: Commun Biol. 2021 Nov 12;4:1287. doi: 10.1038/s42003-021-02823-6 (PMC8590024; doi:10.1038/s42003-021-02823-6)
Supplement: Supplementary file 2 — Supplementary Information [file 42003_2021_2823_MOESM2_ESM.pdf]

Supplementary Information for:

Genome of the estuarine oyster provides insights into climate impact and adaptive plasticity

Ao Li[1,2†], He Dai[3†], Ximing Guo[4†], Ziyan Zhang[1,5,6], Kexin Zhang[1,5,6], Chaogang Wang[1,5,6], Xinxing Wang[1,5,6], Wei Wang[1,5,7], Hongju Chen[3], Xumin Li[3], Hongkun Zheng[3], Guofan Zhang[1,2,7✉] & Li Li[1,5,6,7✉]

[1]CAS and Shandong Province Key Laboratory of Experimental Marine Biology, Center for Ocean Mega-Science, Institute of Oceanology, Chinese Academy of Sciences, Qingdao, China. [2]Laboratory for Marine Biology and Biotechnology, Pilot National Laboratory for Marine Science and Technology, Qingdao, China. [3]Biomarker Technologies Corporation, Beijing, China. [4]Haskin Shellfish Research Laboratory, Department of Marine and Coastal Sciences, Rutgers University, Port Norris, NJ, USA. [5]Laboratory for Marine Fisheries Science and Food Production Processes, Pilot National Laboratory for Marine Science and Technology, Qingdao, China. [6]University of Chinese Academy of Sciences, Beijing, China. [7]National and Local Joint Engineering Key Laboratory of Ecological Mariculture, Institute of Oceanology, Chinese Academy of Sciences, Qingdao, China. [†]These authors contributed equally: A. Li, H. Dai and X. Guo. ✉email: lili@qdio.ac.cn and gzhang@qdio.ac.cn

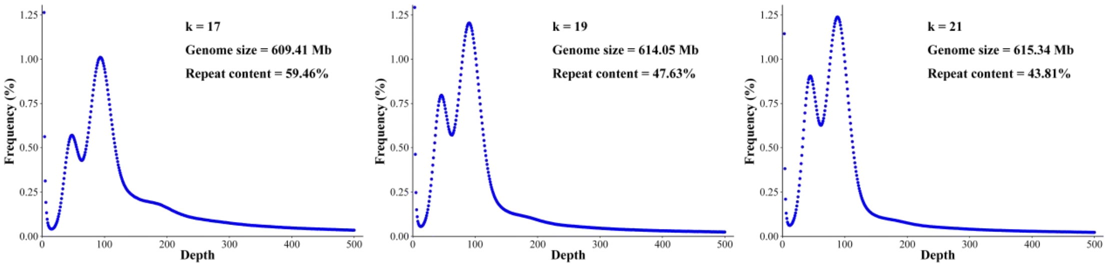

Supplementary Fig. 1 | Distribution of k-mer (k = 17, 19 and 21) frequency in the sequencing reads used to estimate genome size.

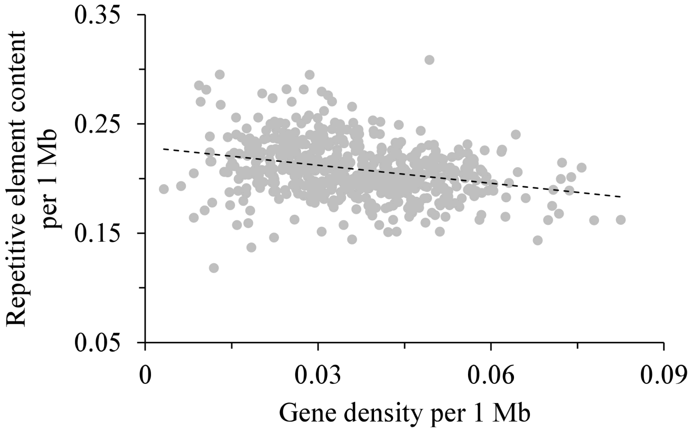

Supplementary Fig. 2 | Negative correlation between gene density and repetitive element content per 1 Mb across the estuarine oyster genome. The trendline indicates fitted line of Pearson correlation.

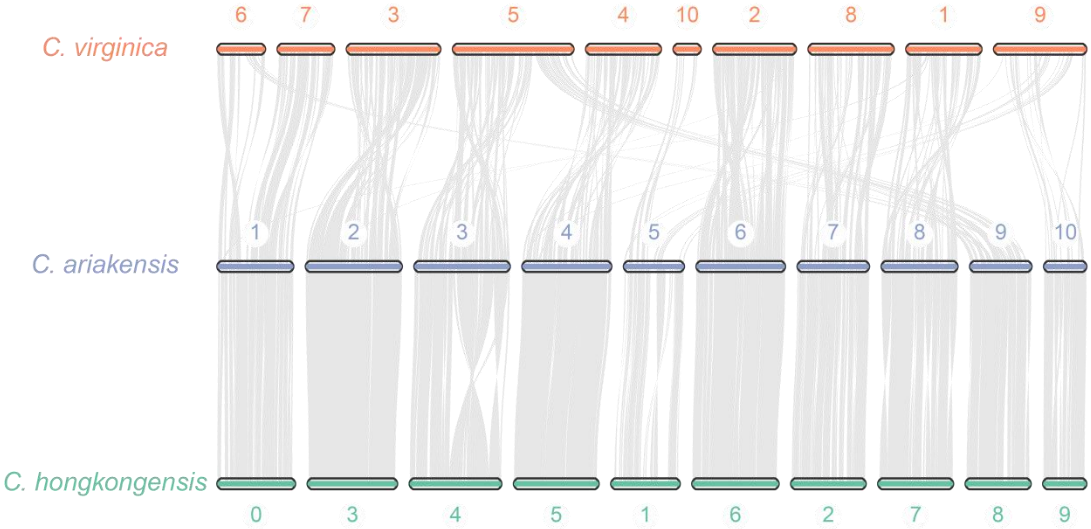

Supplementary Fig. 3 | Comparison of macrosynteny between *C. ariakensis* and two other oyster species living in low-salinity estuaries, *C. hongkongensis* and *C. virginica*.

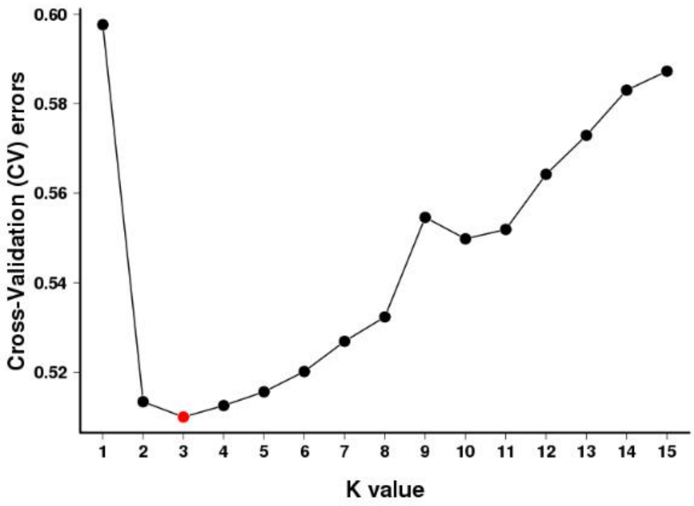

Supplementary Fig. 4 | Cross-validation error plot for populations of the estuarine oyster.

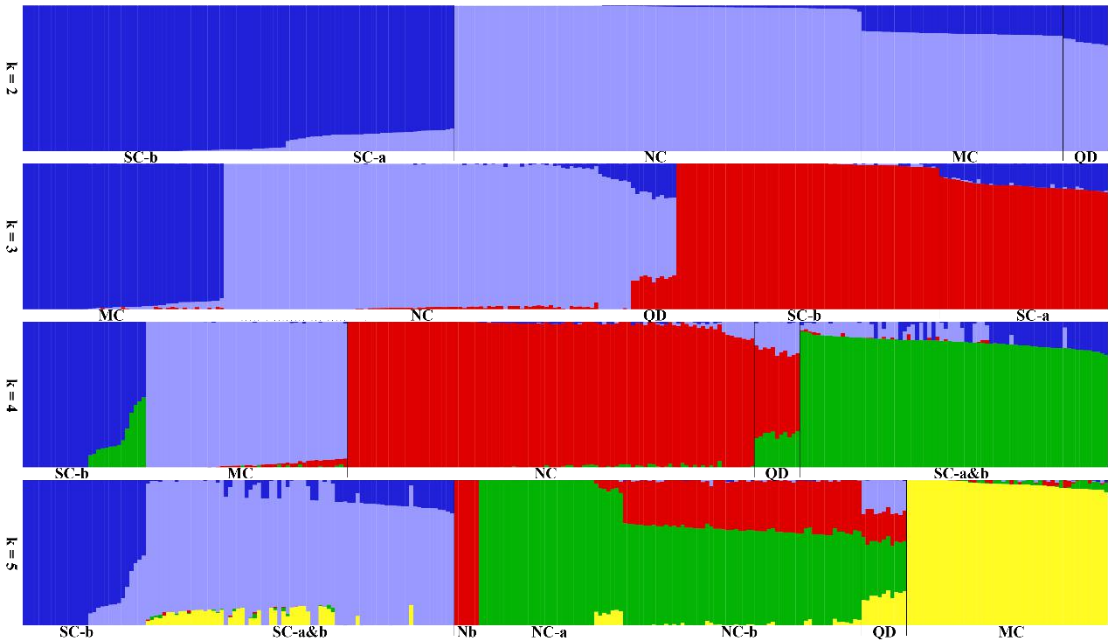

Supplementary Fig. 5 | Model-based clustering analysis of estuarine oyster populations with ADMIXTURE under different number of groups (K = 2, 3, 4 and 5). SC: southern China. NC: northern China (Nb indicates NC-b). MC: middle China. QD: Qingdao population.

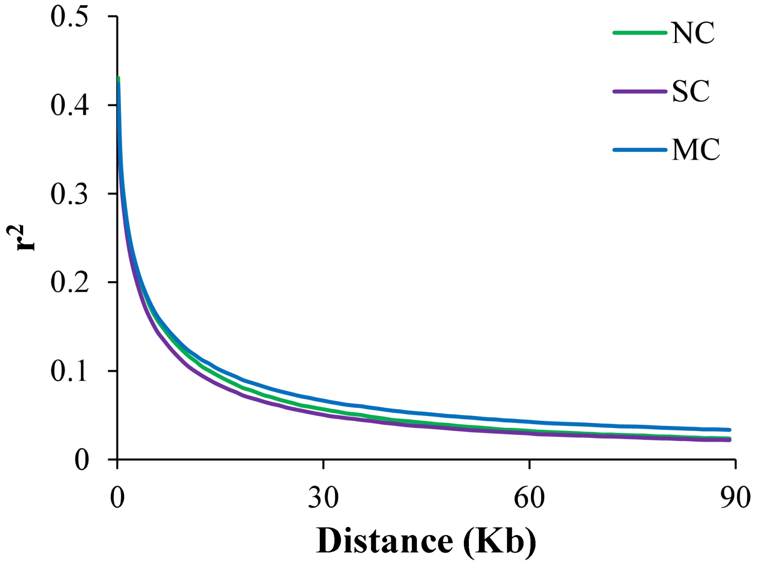

Supplementary Fig. 6 | Decay of linkage disequilibrium in northern (NC), middle (MC) and southern (SC) populations of the estuarine oyster.

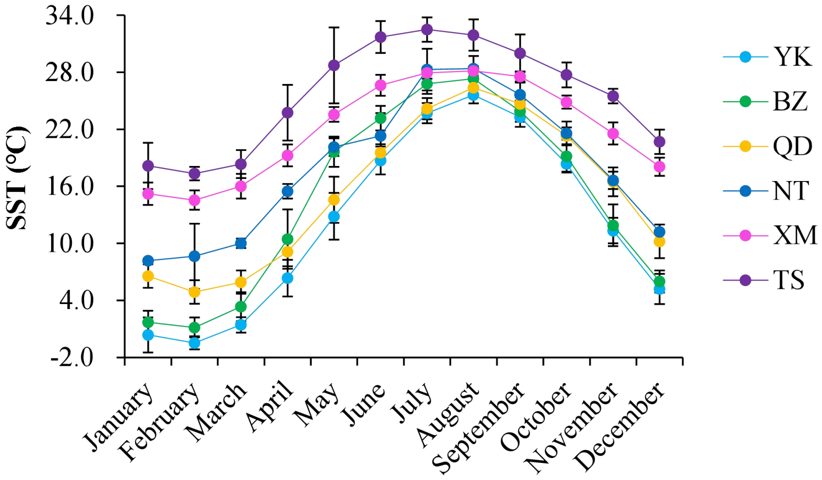

Supplementary Fig. 7 | Monthly average sea surface temperature (SST) from satellite remote sensing data of six sampling sites from each of six subpopulations during 2000 to 2017. Error bars represent SEM values.

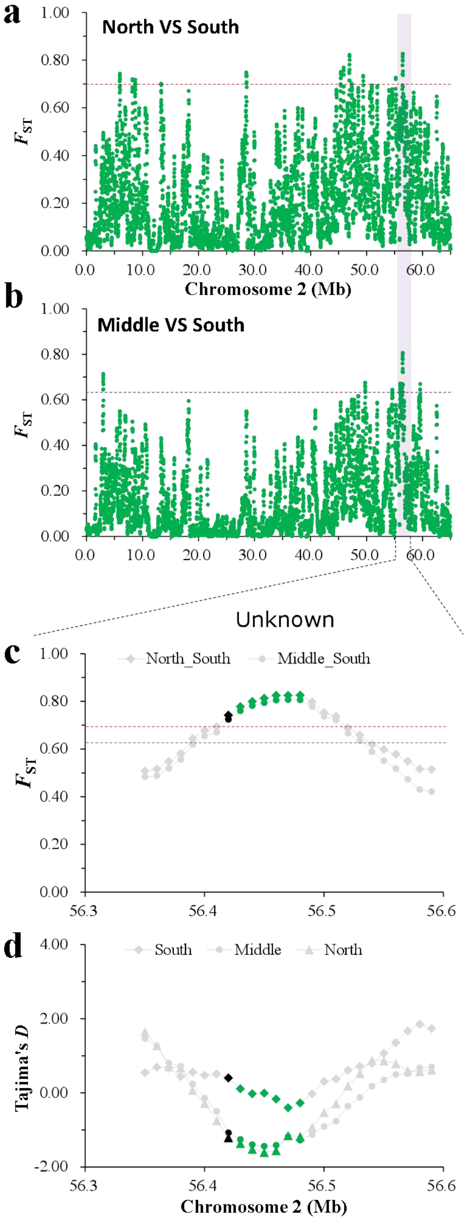

Supplementary Fig. 8 | Signatures of selection along chromosome 2 in northern, middle and southern populations of the estuarine oyster. a and **b**, Selective sweep signatures identified by top 1% F_{ST} value (dash lines) between northern and southern (**a**), and between middle and southern populations (**b**). **c**, F_{ST} values surrounding selective regions om chromosome 2 between population pairs. **d**, Tajima's *D* surrounding selective regions on chromosome 2 in northern, middle and southern populations of *C. ariakensis*. Noncoding regions were marked with black, and coding regions were green.

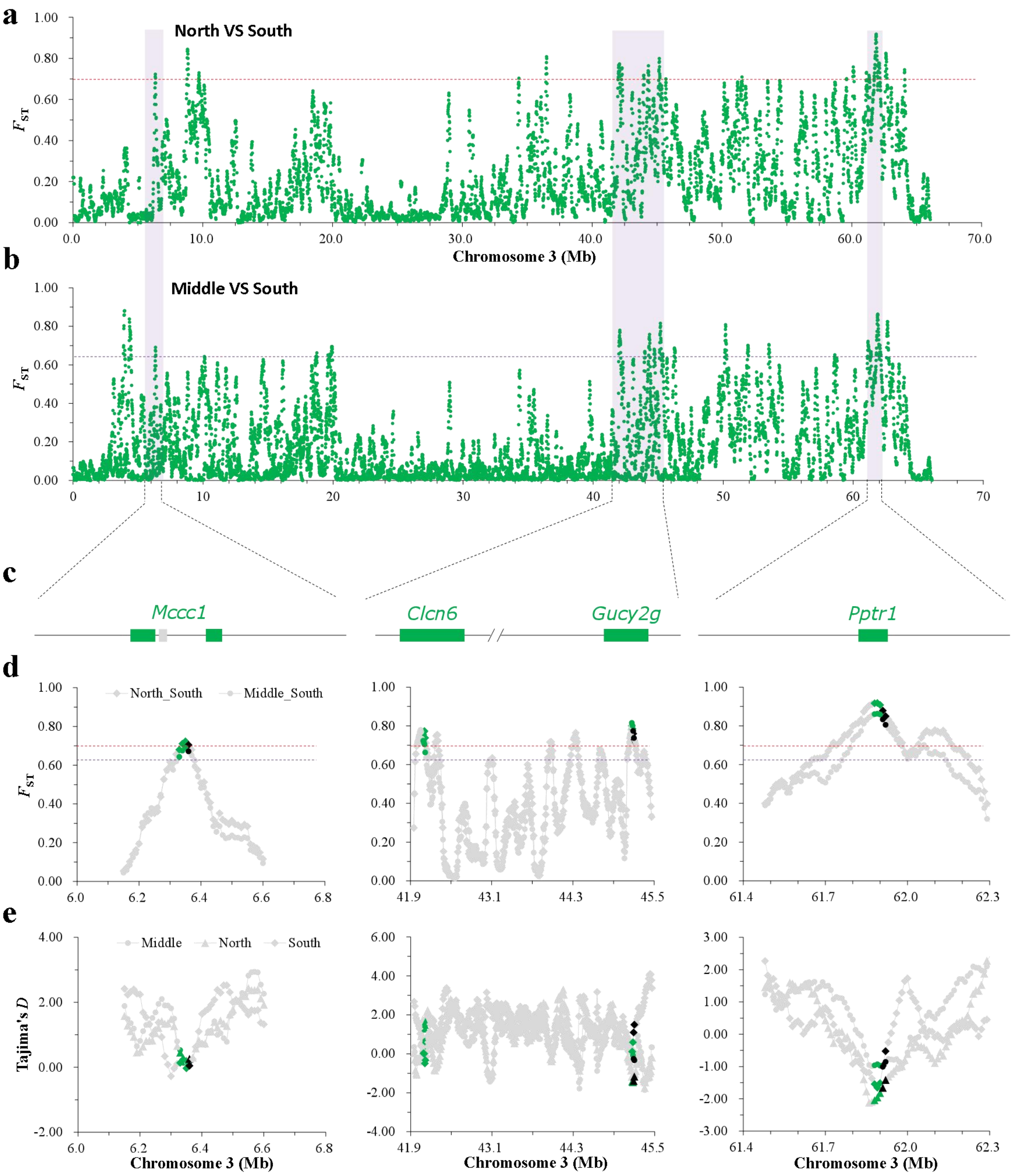

Supplementary Fig. 9 | Signatures of selection along chromosome 3 in northern, middle and southern populations of the estuarine oyster. a and **b**, Selective sweep signatures identified by top 1% F_{ST} value (dash lines) between northern and southern (**a**), and between middle and southern populations (**b**). **c**, Models of annotated genes located within selective regions along chromosome 3. **d**, F_{ST} values surrounding selective regions on chromosome 3 between population pairs. **e**, Tajima's *D* surrounding selective regions on chromosome 3 in northern, middle and southern populations. Noncoding regions were marked with black, and coding regions were green.

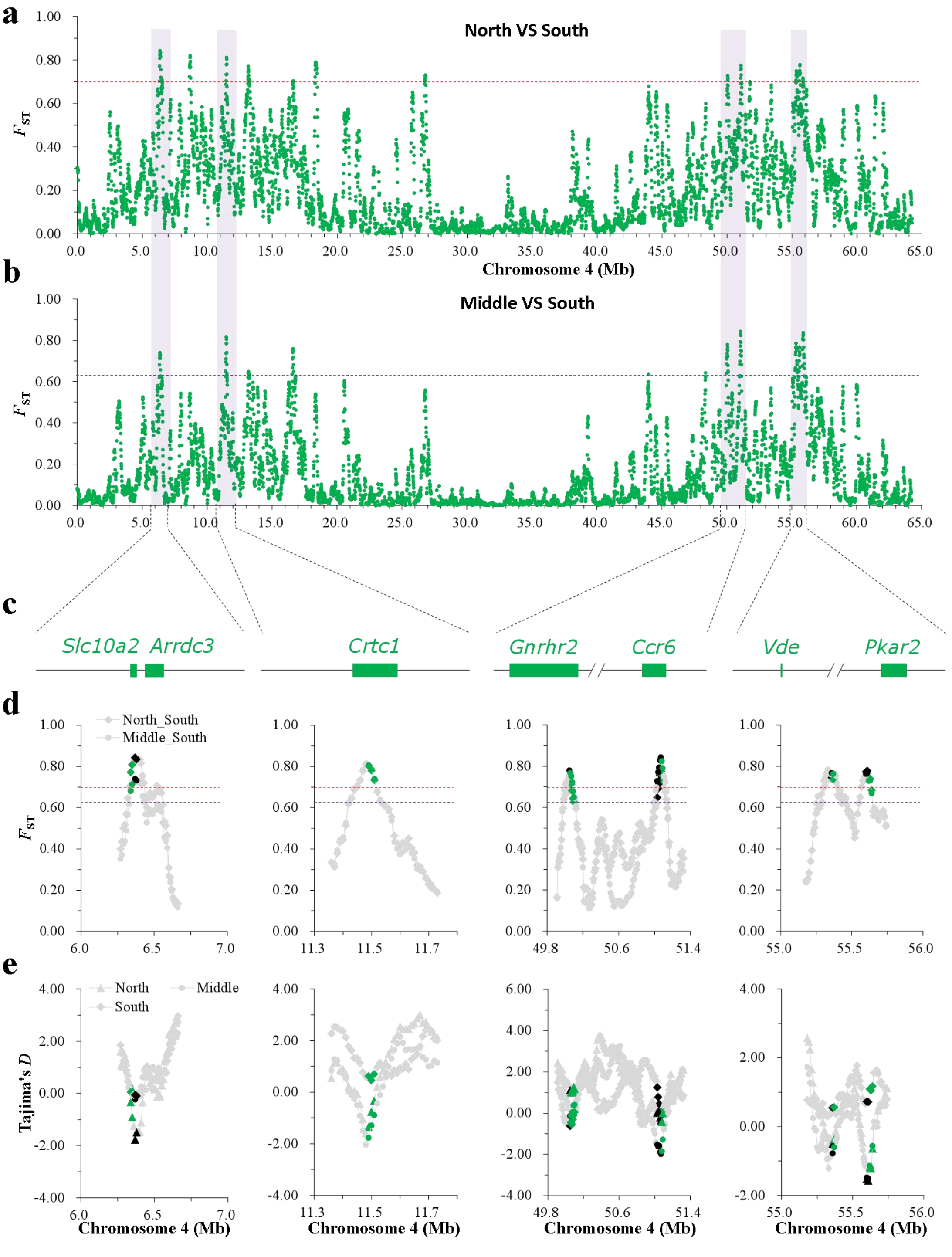

Supplementary Fig. 10 | Signatures of selection along chromosome 4 in northern, middle and southern populations of the estuarine oyster. a and **b**, Selective sweep signatures identified by top 1% F_{ST} value (dash lines) between northern and southern populations (**a**), and between middle and southern populations (**b**). **c**, Models of annotated genes located within selective regions along chromosome 4. **d**, F_{ST} values surrounding selective regions on chromosome 4 between population pairs. **e**, Tajima's *D* surrounding selective regions on chromosome 4 in northern, middle and southern populations. Noncoding regions were marked with black, and coding regions were

green.

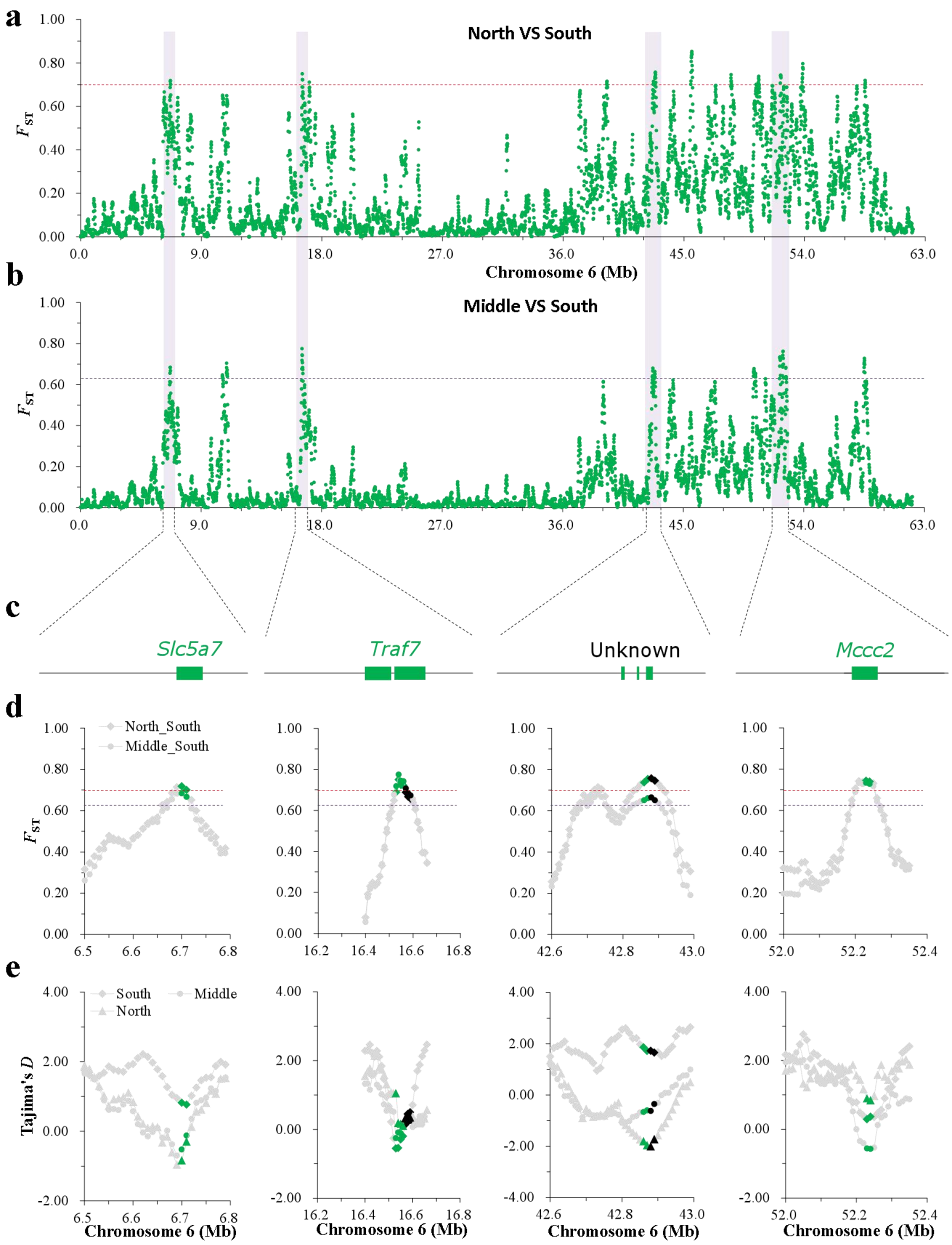

Supplementary Fig. 11 | Signatures of selection along chromosome 6 in northern, middle and southern populations of the estuarine oyster. a and **b**, Selective sweep signatures identified by top 1% F_{ST} value (dash lines) between northern and southern (**a**), and between middle and southern populations (**b**). **c**, Models of annotated genes located within selective regions along chromosome 6. **d**, F_{ST} values surrounding selective regions on chromosome 6 between population pairs. **e**, Tajima's *D* surrounding selective regions on chromosome 6 in northern, middle and southern populations. Noncoding regions were marked with black, and coding regions were green.

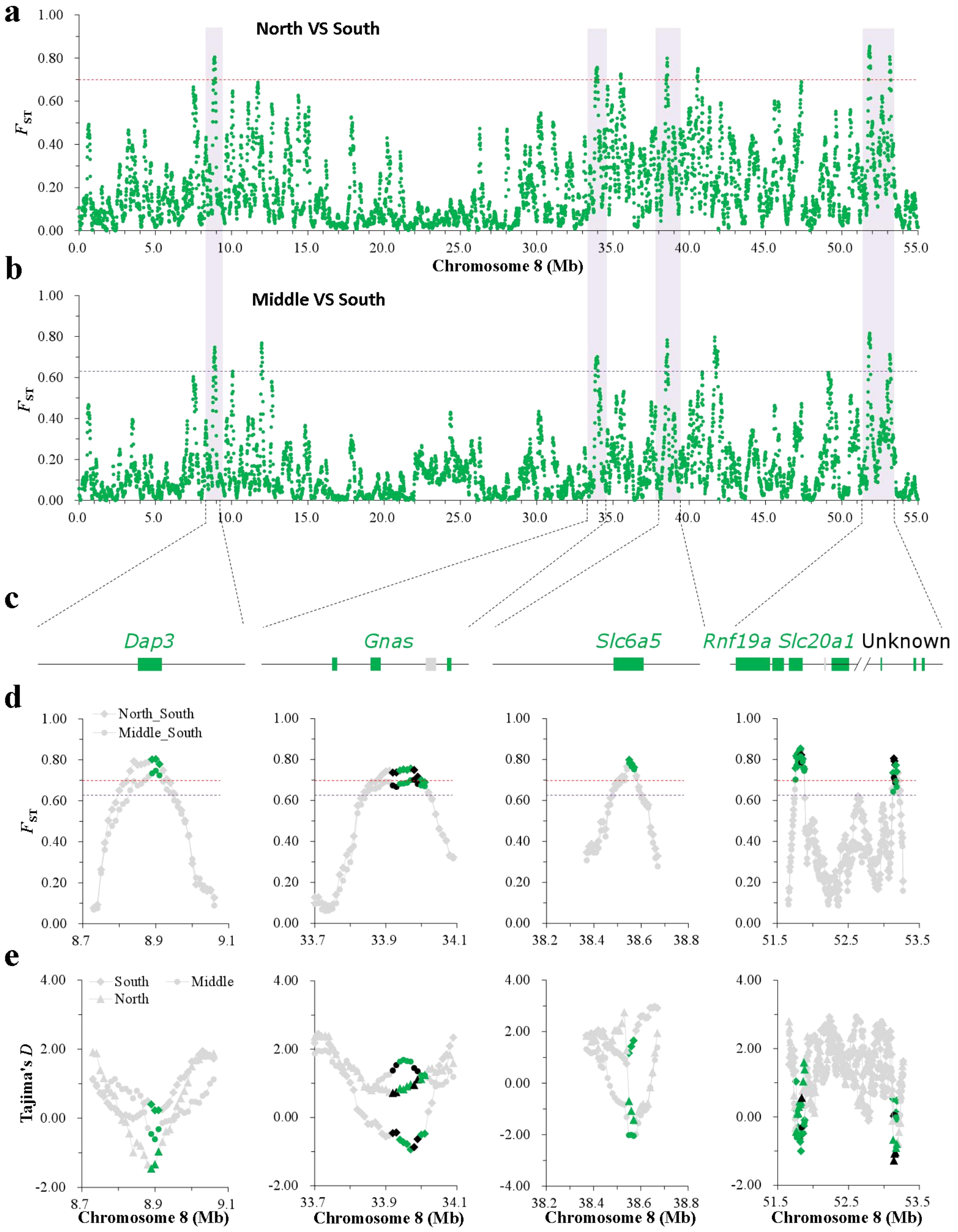

Supplementary Fig. 12 | Signatures of selection along chromosome 8 in northern, middle and southern populations of the estuarine oyster. a and **b**, Selective sweep signatures identified by top 1% F_{ST} value (dash lines) between northern and southern (**a**), and between middle and southern populations (**b**). **c**, Models of annotated genes located within selective regions along chromosome 8. **d**, F_{ST} values surrounding selective regions on chromosome 8 between population pairs. **e**, Tajima's *D* surrounding selective regions on chromosome 8 in northern, middle and southern populations. Noncoding regions were marked with black, and coding regions were green.

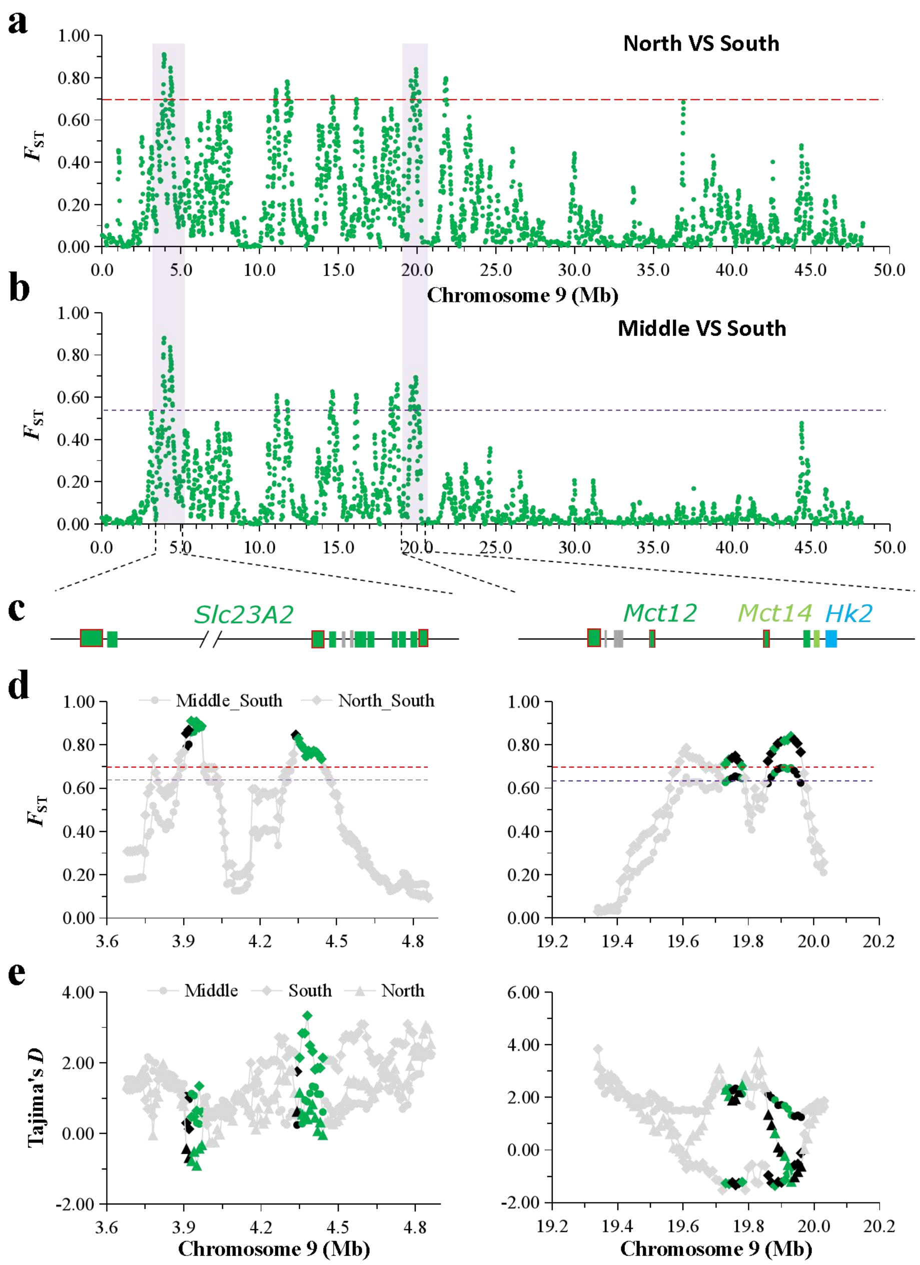

Supplementary Fig. 13 | Signatures of selection along chromosome 9 in northern, middle and southern populations of the estuarine oyster. a and **b**, Selective sweep signatures identified by top 1% F_{ST} value (dash lines) between northern and southern (**a**), and between middle and southern populations (**b**). **c**, Models of annotated genes located within selective regions along chromosome 9. Models with red lines are responsive to temperature and salinity challenges. **d**, F_{ST} values surrounding selective regions on chromosome 9 between population pairs. **e**, Tajima's *D* surrounding selective regions on chromosome 9 in northern, middle and southern populations. Noncoding regions were marked with black, and coding regions were green.

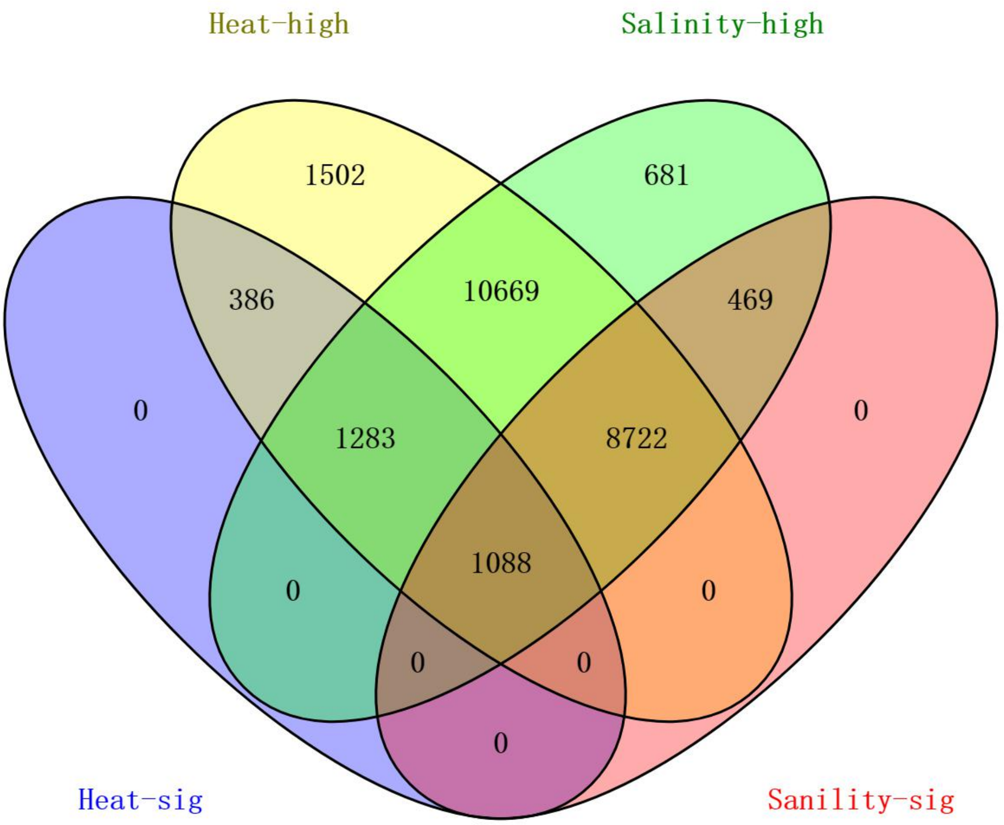

Supplementary Fig. 14 | Venn diagram of genes expressed in response to high temperature and high salinity stresses in the estuarine oyster. Heat-high and salinity-high indicate genes expressed under elevated temperature (6 hours under 37℃ seawater) and high salinity (7 days under 60‰ seawater) excluding genes with low expression. Heat-sig and salinity-sig indicate genes significantly up- or down-regulated in response to high-temperature and high-salinity stresses, respectively.

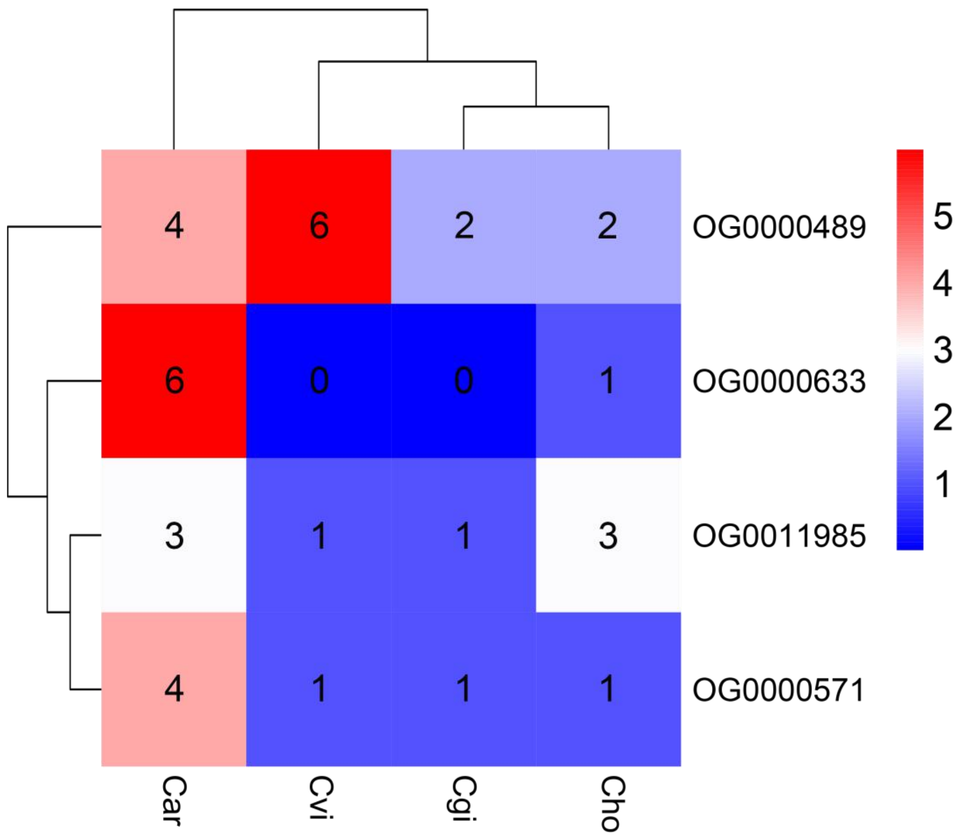

Supplementary Fig. 15 | Copy numbers of orthogroups of *Slc23a2* and *Mct12* gene families in four *Crassostrea* oyster species. OG0000633: uric acid transporter, OG0011985 and OG0000489: purine permease, OG0000571: purine efflux pump. Cvi: *C. virginica*, Car: *C. ariakensis*, Cho: *C. hongkongensis* and Cgi: *C. gigas*. The blue-to-red color bar represents the copy number of the given orthogroup from low to high.

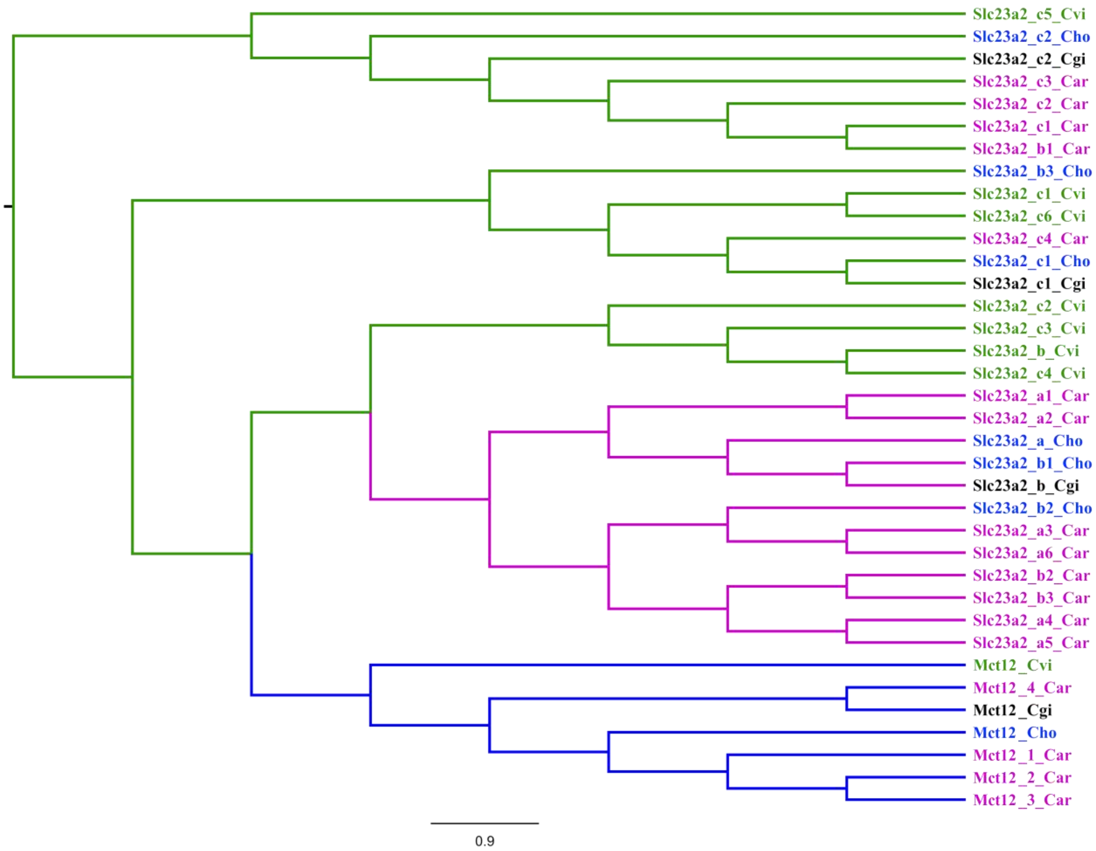

Supplementary Fig. 16 | Phylogenetic tree of orthologs belong to *Slc23a2* and *Mct12* gene families in *Crassostrea* oysters inferred using the Maximum Likelihood (ML) method with 1,000 bootstraps. Green branches indicate most of orthologs (14 of 17) belong to orthogroup OG0000489, purple branches indicate orthologs belong to the orthogoups OG0011985 and OG0000633, and blue branches indicate orthologs belong to the orthogroup OG0000571. Car: *C. ariakensis* (purple), Cho: *C. hongkongensis* (blue), Cgi: *C. gigas* (black), Cvi: *C. virginica* (green).

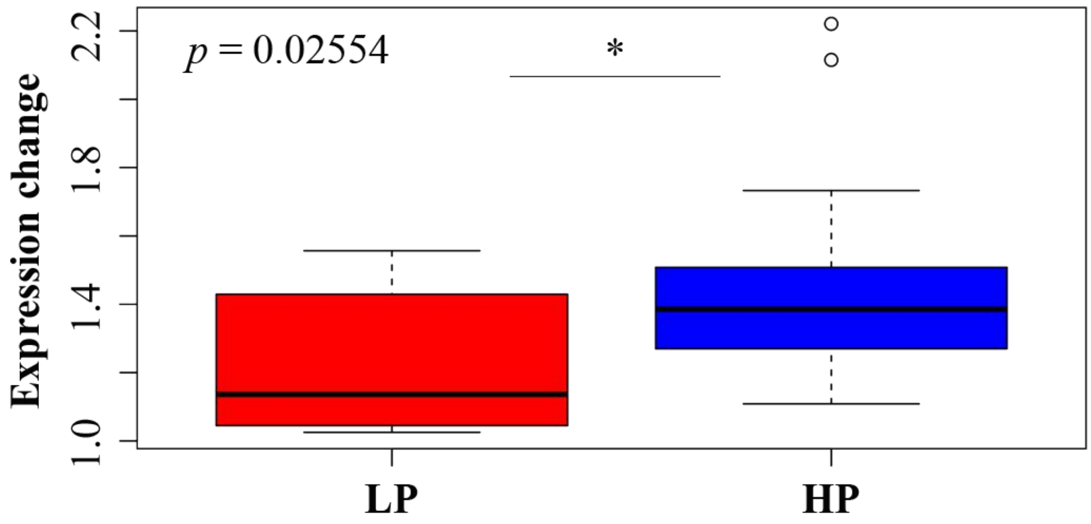

Supplementary Fig. 17 | Expression changes of estuarine oyster genes showing

low plasticity (LP) and high plasticity (HP) when reciprocally transplanted to non-native habitats. Asterisks indicate significant difference (* $p < 0.05$). The data are presented as box plots; the central rectangle spans the first to third quartiles of the distribution, and the 'whiskers' above and below the box show the maximum and minimum estimates. The line inside the rectangle shows the median, the circles represent outliers

Supplementary Table 1. Genome size estimation using the k-mer method. K-mer value used and the estimated genome size of the estuarine oyster *Crassostrea ariakensis*.

	k-mer (k)	Peak (P)	Read Depth (RD)	Estimated genome size (Mb)
Crassostrea ariakensis	19	89	111	614.05

Supplementary Table 2. Order lengths of the 10 assembled estuarine oyster chromosomes.

Chromosome ID	Cluster number	Clustered length (Mb)	Order number	Ordered length (Mb)
Chromosome 1	70	75.18	37	67.70
Chromosome 2	26	67.95	17	67.23
Chromosome 3	21	67.02	15	66.21
Chromosome 4	24	67.07	14	64.51
Chromosome 5	111	65.29	46	53.30
Chromosome 6	17	62.73	11	62.26
Chromosome 7	55	57.97	31	55.28
Chromosome 8	32	56.75	20	55.21
Chromosome 9	24	50.55	15	48.47
Chromosome 10	36	40.81	19	37.37
Total (Ratio %)	416 (89.27)	611.32 (99.58)	225 (54.09)	577.54 (94.47)

Supplementary Table 3. Comparison of the quality among mollusc genomes.

Species	Genome size (Mb)	Gene number	Contig N50 (Mb)	Scaffold N50 (Mb)	BUSCO (%)
Crassostrea ariakensis	613.9	29,631	7.0	62.3	92.2
Crassostrea hongkongensis	610.0	25,675	2.6	55.6	95.8
Crassostrea virginica	684.7	39,493	2.0	75.9	94.5
Crassostrea gigas[a]	647.9	30,724	1.8	58.4	95.6
Crassostrea gigas[b]	586.8	30,078	3.1	60.9	92.5

Chrysomallon squamiferum	444.4	16,917	1.9	30.2	96.6
Acanthopleura granulata	606.9	20,470	1.1	23.9	97.4
Mercenaria mercenaria	1,780	34,283	1.8	91.4	90.5
Scapharca broughtonii	884.5	24,045	1.8	45.0	91.7
Tegillarca granosa	812.6	24,398	0.6	42.6	93.3
Nautilus pompilius	730.6	17,710	1.1	/	93.5
Euprymna scolopes	5,280	29,259	0.0036	3.7	97.0
Helobdella robusta	235.4	23,400	0.052	3.1	/

Supplementary Table 4. Evaluation of the estuarine oyster genome assembly using unigenes assembled from transcriptomes.

Range of length	Total number of transcripts	Total aligned number	Percentage (%)	with >50% sequence in one scaffold		with >90% sequence in one scaffold	
				Aligned number	Percentage (%)	Aligned number	Percentage (%)
All	19,781	19,218	97.15%	18,707	94.57%	18,117	91.59%
>=500	19,614	19,069	97.22%	18,558	94.62%	17,973	91.63%
>=10,00	18,694	18,184	97.27%	17,679	94.57%	17,110	91.53%

Supplementary Table 5. Identity between assembled genome sequences and sequences from Sanger for 10 randomly selected genomic segments.

**ID**	**Primers**	**Length**	**Identity**
Chr02_1	F:TCGTTCAAATCACCTCCCA<br>R:CTGCCTGTCCAAACCCAT	410	98.78%
Chr02_2	F:GACCCTTTTATCCCCACC<br>R:TTCTATGGAGCCACTACCTT	305	98.69%
Chr03	F:CAGAGGCAACTGCGAAAG<br>R:AATGACGGACAGGATGGG	488	98.36%
Chr04	F:TTCGCTGGATTCTGTAAGA<br>R:AATGGGAAGGATTGATGTG	437	98.63%
Chr05_1	F:TTTGGCATAGGTAAAACACGC<br>R:CGCCTCCTTCAAGATACGAT	268	97.76%
Chr05_2	F:ATTGAGAAACAAGCCAAAGGG<br>R:TGCGATGCTCAGTTGTCCTA	583	99.14%
Chr06	F:AACATTCCTCGCACTCAG<br>R:GTACGCTCACCGAAGATAC	594	97.64%

Chr07	F:ACGCTTGCCAAACTCCCA<br>R:TTTCCAGCCCAACCCCTAC	319	97.81%
Chr08	F:GAAAAGAACGGACACCAG<br>R:GAACCAAAGGGGAGACTAA	275	100.00%
Chr09	F:AGGCAATAAGCGACCATAACT<br>R:AACCTGGGTAAGGGAACTGA	494	96.36%

Supplementary Table 6. Functional annotation of genes from the estuarine oyster genome with different databases.

Database	Annotated number	100<=Protein length<300	Protein length>=300	Percentage of the total predicted gene (%)
GO	5739	1641	3995	18.62%
KEGG	10453	2545	7783	33.91%
KOG	15484	3606	11749	50.24%
Pfam	20415	5616	14607	66.23%
Swissprot	14434	3519	10761	46.83%
TrEMBL	29529	10590	18483	95.80%
nr	29534	10585	18491	95.82%
All annotated	29631	10640	18528	96.13%

Supplementary Table 7. Repetitive sequences in the eaturine oyster genome assembly.

**Repeat class**	**Repeat subclass**	**Number of elements**	**Length occupied (bp)**	**Percentage of sequence**
**Retroelement**		540,114	185,898,216	30.28
	LTR-Copia	840	235,392	0.04
	LTR-Gypsy	43,321	22,874,205	3.73
	LTR-DIRS	16,330	6,687,043	1.09
	LTR-other	7,705	3,556,964	0.58
	LARD	239,750	83,986,861	13.68
	LINE	84,775	26,732,170	4.35
	SINE	1,455	301,006	0.05
	Penelope	136,625	36,593,463	5.96
	TRIM	4,901	4,137,166	0.67
	unclassified	4,412	793,946	0.13
**DNA Transposon**		681,904	204,043,881	33.24
	TIR	109,992	25,615,078	4.17
	Helitron	232,234	89,357,475	14.56
	Maverick	3,715	1,426,222	0.23
	MITE	5,041	1,107,524	0.18
	Crypton	224,076	54,503,925	8.88
	unclassified	106,846	32,033,657	5.22
**PotentialHostGene**		1,120	531,739	0.09
**SSR**		1,264	329,934	0.05

**Unknown**	56,725	13,985,686	2.28
**Total with overlap**	1,281,127	404,789,456	65.94
**Total without overlap**	1,281,127	332,399,967	54.14

LTR: long terminal repeats, LINE: long interspersed nuclear elements, SINE: short interspersed nuclear elements.

Supplementary Table 8. Pair-wise fixation statistics (F_{ST}) among 11 populations based resequencing data of 264 wild estuarine oysters.

Population	Site	North				QD	Middle		South		
		DD	YK	BZ	DY		NT	SH	JLJ	ZhJ	TS
North	DD										
	YK	0.0189									
	BZ	0.0207	0.0152								
	DY	0.0232	0.0178	0.0026							
	QD	0.0538	0.0599	0.0436	0.0447						
Middle	NT	0.0439	0.0474	0.0392	0.0410	0.0382					
	SH	0.0434	0.0465	0.0388	0.0404	0.0378	0.0008				
South	JLJ	0.2052	0.2165	0.1914	0.1915	0.1190	0.1346	0.1343			
	ZhJ	0.2076	0.2190	0.1939	0.1941	0.1209	0.1365	0.1362	0.0006		
	TS	0.2529	0.2633	0.2361	0.2366	0.1592	0.1786	0.1785	0.0166	0.0152	
	QZh	0.2720	0.2822	0.2548	0.2552	0.1760	0.1961	0.1961	0.0280	0.0264	0.0173

Supplementary Table 9. Expression level for gene copies of *Slc23a2* and *Mct12* families under heat and high-salinity stresses.

**Orthologs**	**Gene name**	**Gene ID**	**YK_normal**	**YK_high salinity**	**BZ_normal**	**BZ_heat stress**
OG0000571	*Mct14_1*	EVM0027441	1.807131	0.6120274	0	0
	Mct14_2	EVM0028048	4.400541	2.0429662	0	0
	Mct14_3	EVM0020039	4.2903816	1.3717096	0	0
	Mct14_4	EVM0020452	0	0	0	0
OG0011985	*Slc23a2_a1*	EVM0005132	12.7053952	0.8016414	11.0552222	22.3916018
	Slc23a2_a2	EVM0017864	1.7938348	0.907227	9.745423	2.7643728
	Slc23a2_a3	EVM0001683	0	0	0	0.4312714
	Slc23a2_a4	EVM0003333	0	0	0	0
	Slc23a2_a5	EVM0008135	0	0	0	0
	Slc23a2_a6	EVM0016791	0	0	0	0.34522
OG0000489	*Slc23a2_c1*	EVM0004423	0.2150472	0	0.3334546	0
	Slc23a2_c2	EVM0006462	7.0648708	2.8127208	7.346915	3.333624
OG0000633	*Slc23a2_b1*	EVM0013745	0	0	0.181059	0
	Slc23a2_b2	EVM0022380	0	0	0	0

Supplementary Table 10. Summary for three rounds of correction by Pilon.

**Genome_size**	**SNP**	**Insert**	**Delete**	**Error ratio (%)**

777,213,952	442,861	393,016	2,378,169	0.41
779,067,884	98,576	26,236	59,702	0.02
779,041,778	29,647	6,240	12,946	0.01

Supplementary Table 11. Summary for the final round of correction by both Racon and Pilon.

Correction	Contig Number	Genome size	Contig N50	Contig N90	Contig Maximum	GC(%)
Before Racon	1,777	773,515,823	5,384,006	270,489	20,131,303	33.36
After Racon	1,777	777,213,952	5,454,258	274,035	20,452,959	33.34
After Pilon	1,777	779,025,439	5,465,454	274,941	20,491,775	33.39

Supplementary Table 12. Weight value for gene prediction indicated by homology, de novo prediction and mRNA transcripts.

Method	Software	Weight value
Homology-based	GeMoMa	50
RNAseq	PASA	50
Ab initio	Genscan	0.3
	AUGUSTUS	0.3
	SNAP	0.3
	GeneID	0.3
	GlimmerHMM	0.3

Supplementary Movie 1 | Dynamics of average monthly sea surface temperature (SST) and ocean currents along Chinese coastlines. Data for SST and ocean currents were downloaded from MODIS (Jan 2003 to Dec 2019) and OSCAR (Jan 2001 to Dec 2019), respectively.
