# Supplementary material for: Genome of the estuarine oyster provides insights into climate impact and adaptive plasticity
Source: Commun Biol. 2021 Nov 12;4:1287. doi: 10.1038/s42003-021-02823-6 (PMC8590024; doi:10.1038/s42003-021-02823-6)
Supplement: Supplementary file 3 — Description of Additional Supplementary Files [file 42003_2021_2823_MOESM3_ESM.pdf]

Description of Additional Supplementary Files

File name: Supplementary Movie 1

Description: Dynamics of average monthly sea surface temperature (SST) and ocean currents along Chinese coastlines. Data for SST and ocean currents were downloaded from MODIS (Jan 2003 to Dec 2019) and OSCAR (Jan 2001 to Dec 2019), respectively.

File name: Supplementary Data 1-6

Description:

Supplementary Data 1: Assessment for the completeness and accuracy of gene coverage using Benchmarking Universal Single-Copy Orthologs (BUSCO).

Supplementary Data 2: Synteny analysis for genomes between C. ariakensis and C. hongkongensis.

Supplementary Data 3: Synteny analysis for genomes between C. ariakensis and C. virginica.

Supplementary Data 4: Statistics of whole-genome resequencing of 264 wild estuarine oysters.

Supplementary Data 5: Annotation of genes from regions with selection signals.

Supplementary Data 6: Accession numbers for oysters used in genome sequencing, whole-genome resequencing and transcriptomic analysis.

File name: Supplementary Data 7

Description: The source data underlying (a) Figure 3a, (b) Figure 3c, (c) Figure 3d, (d) Figure 3e, (e) Figure 5b and (f) Figure 5c.
