# Supplementary figures and images for: Genome of the estuarine oyster provides insights into climate impact and adaptive plasticity
Source: Commun Biol. 2021 Nov 12;4:1287. doi: 10.1038/s42003-021-02823-6 (PMC8590024; doi:10.1038/s42003-021-02823-6)

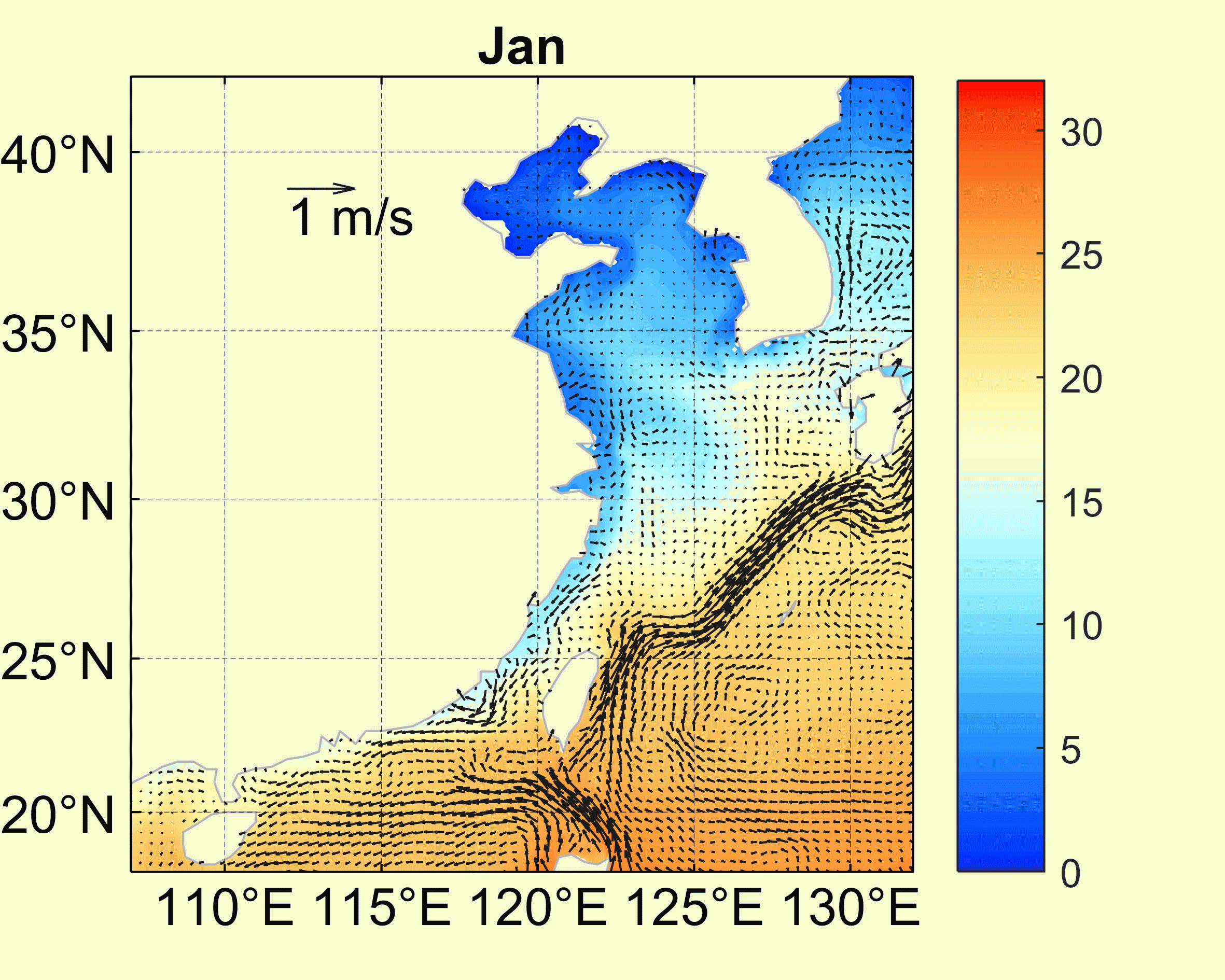

Supplement: Supplementary file 4 — Supplementary Movie 1 [file 42003_2021_2823_MOESM4_ESM.gif]
